# Curing modes affects micro-tensile bond strength and durability of dual curing resin cements to dentin

**DOI:** 10.3389/fbioe.2024.1511099

**Published:** 2025-01-09

**Authors:** Zimeng Li, Xiaoyuan Yan, Negin Toufani, Hidehiko Sano, Jiale Fu

**Affiliations:** ^1^ Liaoning Provincial Key Laboratory of Oral Disease, School and Hospital of Stomatology, China Medical University, Shenyang, China; ^2^ Department of Restorative Dentistry, Division of Oral Health Science, Faculty of Dental Medicine, Hokkaido University, Sapporo, Japan; ^3^ Liaoning Provincial Key Laboratory of Oral Disease, Department of Dental Materials Science, School and Hospital of Stomatology, China Medical University, Shenyang, China

**Keywords:** dual curing resin cement, universal adhesive, light-curing, self-curing, bonding durability

## Abstract

**Background:**

To evaluate the effects of curing modes on the micro-tensile bond strength and durability of 6 different dual curing resin cements to dentin.

**Methods:**

84 human molars were divided into 7 groups (n = 12), and were further distributed into two subgroups according to the two curing modes: light-curing (L) and self-curing (D) (n = 6). The 6 dual-cured resin cements were: DU (Duolink, Bisco), TC (TheraCem, Bisco), MS (Multilink Speed, Ivoclar), N3 (Nexus 3 Universal, Kerr), R2 (RelyX U200, 3M) and RU (RelyX Ultimate, 3M). The universal adhesives were: All Bond Universal (Bisco); Adhesive Universal Vivapen (Ivoclar); Optibond Versa Adhesive (Kerr); Scotch Bond Universal Adhesive (3M). RU was placed directly on processed adhesive layer without light-curing, set as group 7. Micro-tensile bond strength test was carried out after 37°C water storage for 24 h, 3 months and 6 months, respectively. Three-way ANOVA and Games-Howell test were performed by SPSS 19.0 (α = 0.05).

**Results:**

The Three-way ANOVA indicated that bonding performance was significantly affected by curing mode (p = 0.000, F = 1,237.510), resin cement (p = 0.000, F = 59.507) and storage time (p = 0.000, F = 97.888). The interaction of these three factors was significant (p = 0.017, F = 2.071).

**Conclusion:**

The bonding strength and durability of dual curing resin cements were significantly improved by light-curing process. In addition, the bonding strength of resin cements with different experimental condition was material depended. Among all tested resin cements, N3 exhibits the most favorable outcomes.

**Clinical Relevance:**

A dual curing procedure is recommended in clinical application. Among all tested resin cements, N3 exhibits the most favorable outcomes.

## 1 Introduction

Riding on the extensively operated restorations on structurally compromised teeth, concerns have been expressed about the bond between restorative materials and enamel/dentin surface. Bonding strength is extensively determined by cementation procedure, which can be roughly divided into two types: conventional cementation and adhesive luting (Perdigão et al., 2021). Compared to conventional cementation relied on simply mechanical bonding, adhesive luting–which is mainly performed by resin cement - profits from a set of mechanical, micromechanical and chemical bonding adhesions (Sakaguchi et al., 2019).

To date, the resin cement can be categorized into light-curing, self-curing and dual curing per as different curing modes. Compared to self-curing resin cements, light-curing resin cements exhibit higher bonding strength and increased durability (Aldhafyan et al., 2021; Kim et al., 2020). However, the clinical performance of light-curing resin cements can be significantly compromised when light access is limited. For instance, the luting between opaque indirect restorative materials and dentin is adversely affected by the opacity of the materials, resulting in inadequate polymerization and consequently insufficient bonding strength (Pishevar et al., 2019; Zhou et al., 2020). Therefore, dual-curing resin cement has been introduced with both photo-initiator and self-curing initiator. This ensures complete polymerization in particular areas where curing light cannot penetrate (Mazzitelli et al., 2023). Nevertheless, there is some doubt about whether a self-initiated luting system can provide sufficient bonding strength, given that light curing is essential for achieving the maximum degree of polymerization (Heboyan et al., 2023; Aguiar et al., 2010).

Although both light-curing and self-curing modes are available in dual curing resin cement, the utilization of self-curing alone is not recommended. In clinical practice, thickness of 2–3 mm is preferred for dual curing cement to still provide significant luting ability due to light attenuation through opaque resin cement or ceramic restorations (Kilinc et al., 2011; Akgungor et al., 2005; Lu et al., 2017). The self-curing components in dual curing resin cement can compensate for this deficiency in polymerization (Heboyan et al., 2023). However, according to previous studies, light access had been emphasized as an indispensable factor during resin cement polymerization (He et al., 2024). The presence of light has been identified as a potential means of preventing resin degradation and preserving relevant properties of dual curing resin cement (Aldhafyan et al., 2021). Otherwise, incomplete polymerization may lead to the elution of unexpected substances such as residual unreacted monomers and byproducts, which is more likely to occur with self-curing luting systems, resulting in adverse effects on the surrounding dental tissues (Kondo et al., 2024; Diemer et al., 2021; Aldhafyan et al., 2022a). Prior studies have demonstrated that the dual curing resin cement exhibits significantly greater cytotoxicity when only chemically cured (Schmid-Schwap et al., 2009; D’Alpino et al., 2017).

More new products have been released in recent years aiming at a more reliable dual curing mode that eliminates the light-curing step, thus reduce chairside time. For example, RelyX Ultimate is formulated by a component that triggers the self-curing of SBUs. However, the bonding performance and durability of dual curing self-etching resin cement omitted light-curing was rarely reported.

This present study was designed to investigate the curing modes (light-curing/self-curing) on the micro-tensile bond strength and durability (24 h, 3 m and 6 m) of 6 dual curing resin cements to dentin. The null hypotheses tested as follow: (i) each resin cement performs the same bonding performance; (ii) the curing modes and storage condition does not affect the bonding strength of different resin cements.

## 2 Materials and methods

6 resin cement were evaluated in this study: (1) Duolink(DU), (2) TheraCem(TC), (3) Multilink Speed(MS), (4) Nexus3(N3), (5) RelyX U200(R2), (6) RelyX Ultimate(RU). 4 universal adhesives were used: (1) All Bond Universal(ABU), (2) Adhesive Universal Vivapen(AUV), (3) Optibond Versa Adhesive(OVA), (4) Scotch Bond Universal Adhesive(SBU). The compositions of these cements as indicated by the manufacturers are listed in Table 1. The associated composition and manufacturers’ instructions of experimental adhesives are shown in Table 1. The associated composition and manufacturers’ instructions of experimental adhesives are shown in Table 2.

**TABLE 1:** Overview of all resin cements employed.

Cements	Type	Based composite
TheraCem (Bisco, United States)	Dual-cure, self-etching, self-adhesive resin cement	Portland Cement, Ytterbium w/Barium Glass, BisGMA^a^; Catalyst: 10-MDP^b^, HEMA^c^, Tert-butyl Perbenzoate
Multilink Speed (Ivoclar Vivadent, Liechtenstein)	Self-adhesive, self-curing cement with light-curing option	UDMA^d^, TEGDMA^e^; Catalyst: UDMA, TEGDMA, methacrylated phosphoric acid ester, PEGDMA^f^, benzoyl peroxide
RelyX U200 (3M ESPE, Germany)	Dual-cure, self-adhesive resin cement	Methacrylate monomers containing phosphoric acid groups, methacrylate monomers, silanated fillers, initiator components, stabilizers, rheological additives Catalyst: methacrylate monomers, alkaline (basic) fillers, silanated fillers, initiator components, stabilizers, pigments, rheological additives
Duo-Link (Bisco, United States)	Dual-cure adhesive resin cement	Bis-GMA, TEGDMA, UDMA glass filler (filler content: 61.9 ± 0.43 wt%)
Nexus 3(Kerr, Germany)	Dual-cure adhesive resin cement	Methacrylate Ester monomers, inert mineral fillers, activators, stabilizers and radiopaque agent. Nexus 3 is a mine-free initiator system
RelyX Ultimate (3M ESPE, Germany)	Dual-cure adhesive resin cement	Methacrylate monomers, radiopaque, silanated fiilers, initiator components, stablizers, rheological additives. Catalyst paste: Methacrylate monomers, radiopaque alkaline (basic) fillers, initiator components, stabilizers, pigments, rheological additives, fluorescence dye, dark-cure activator for Scotchbond Universal Adhesives

^a^ Bis-GMA, bisphenol A-glycidyl methacrylate.

^b^ 10-MDP, 10-methacryloyloxydecyl dihydrogen phosphate.

^c^ HEMA, hydroxyethylmethacrylate.

^d^ UDMA, urethane dimethacrylate.

^e^ TEGDMA, triethyleneglycol dimethacrylate.

^f^ PEGDMA, polyethylene glycol dimethacrylate.

**TABLE 2:** List of all adhesives used and their manufacturers’ instructions.

Adhesives	Compositions	Instructions
All Bond Universal (Bisco, United States)	10-MDP^a^, Bis-GMA^b^, HEMA^c^, ethanol, water, initiators	1. Apply two separate coats of adhesive, scrubbing the preparation with a micro-brush for 10–15 s per coat. Do not light cure between coats 2. Evaporate excess solvents by thoroughly air-drying with an air syringe for at least 10 s, there should be no visible movement of the material. The surface should have a uniform glossy appearance 3. Light cure for 10 s
Adhese Universal Vivapen (Ivoclar Vivadent, Liechtenstein)	Bis-GMA, HEMA, phosphoric acid ester monomers, decandiol dimethacrylate, ethanol, water, camphorquinone, ethyl p-dimethyl aminobenzoate, silica filler	1. Adhesive applied to air-dried tooth surface with rubbing action for 20 s 2. Medium air pressure applied to surface for 5 s 3. Adhesive photo-polymerized for 10 s
Optibond Versa Adhesive (Kerr, Germany)	PRIMER: GPDM ^d^, hydrophilic comonomers, water/ethanol, acetone ADHESIVE: resin monomers, inorganic fillers, ethanol	1. Apply PRIMER to dentin using scrubbing motion for 20 s 2. Air thin with medium air pressure for 5 s 3. Shake ADHESIVE bottle briefly. Apply to enamel/dentin surface using light brushing motion for 15 s 4. Air thin with medium air pressure and then strong air for at least 5 s 5. Light cure for 10 s
Scotch Bond Universal Adhesive (3M ESPE, Germany)	10-MDP, HEMA, ethanol, water, dimethacrylate resins, phosphate monomer, methacrylate-modified polyalkenic acid copolymer, filler, initiators, silane	1. Apply the adhesive to the entire preparation with a microbrush and rub it in for 20 s 2. Direct a gentle stream of air over the liquid for about 10 s until the solvent is evaporated completely 3. Light-cure for 10 s

^a^ 10-MDP, 10-methacryloyloxydecyl dihydrogen phosphate.

^b^ Bis-GMA, bisphenol A-glycidyl methacrylate.

^c^ HEMA, hydroxyethylmethacrylate.

^d^ GPDM, glycerol phosphate dimethacrylate.

### 2.1 Specimen preparation

A total of 84 extracted human molars that had been extracted for reasons not related to this study were collected and stored in distilled water. The teeth were collected under a protocol approved by the Institutional Review Board of School and Hospital of Stomatology, China Medical University, Shenyang, China (G2018026). The occlusal surfaces of teeth were grinded smooth and flat by a plaster model trimmer (MT3 18080000; Renfert) under water cooling, until the dentin is fully imposed and achieved the maximum of dentin exposure without reaching the pulp chamber. The regions to be bonded were later polished with wet # 600–grit silicon carbide (SiC) paper under running water for 1 min. Air-drying was applied, leaving the dentin surface with no visible moisture.

**FIGURE 1:**
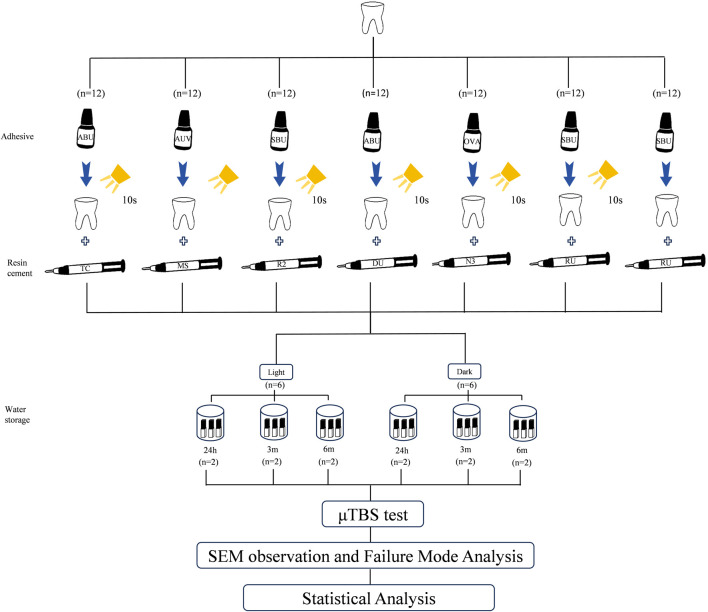
Brief experimental design.

The specimens were randomly divided into 7 groups in accordance with different resin cement introduced above; group 1: TC, group 2: MS, group 3: R2, group 4: DU, group 5: N3 and group 6: RU. In group 7: RU (–S), RU was placed directly on processed adhesive surface without undergoing light-curing. In group 1–6, dual curing resin was placed on the light-curing (DemiPlus; Kerr, United States, 2000 mW/cm2) adhesive layer above their corresponding prepared teeth. Each group was then further distributed into 2 subgroups according to two curing modes (n = 7): light-curing subgroup (/L) and self-curing subgroup (/D), resulting in a total of 14 subgroups. The universal adhesives used in each group were from the same manufacturers as resin cements as follows: ABU for group 1 (TC) and group 4 (DU), AUV for group 2 (MS), OVA for group 5(N3), and SBU for group 3 (R2), group 6 (RU) and group 7 (RU (–S)). A summary of the experimental groups is demonstrated in Table 3, and the experimental process is depicted in Figure 1.

**TABLE 3:** Experimental groups.

Group number	Code designation	Adhesive
1	TC/L^a^, TC/D^b^	ABU
2	MS/L, MS/D	AUV
3	R2/L, R2/D	SBU
4	DU/L, DU/D	ABU
5	N3/L, N3/D	OVA
6	RU/L, RU/D	SBU
7	RU–S/L^c^, RU–S/D^d^	SBU

^a^ /L: resin cement was light-cured.

^b^ /D: resin cement was chemically cured.

^c^ –S/L: adhesive was self-cured and resin cement was light-cured.

^d^ –S/D: adhesive was self-cured and resin cement was chemically cured.

For light-curing mode (/L), 3 layers of resin cement, each layer with approximately 2 mm thickness, were subjected to light-curing for 20 s (DemiPlus, Kerr, United States, 2000 mW/cm2). For self-curing mode (/D), resin cement with a thickness of 6 mm in total was placed above the adhesive layer, and the specimens were then stored in a dark box at 37°C for 48 h for complete chemical-curing. Operations were carried out according to the manufacturers’ instructions. To reduce the effect of surrounding light on the pre-pared specimens, all the procedures were performed under dimmed light.

After water storage (Polystat; Cole-Parmer, United States) at 37°C for 24 h, each sample was cross-sectioned longitudinally with a low-speed diamond saw (SYJ–150; Kejing, China) to acquire beam-shaped specimens with an approximate surface area of 1 × 1 mm^2^. The specimens were randomly distributed into 3 groups based on different water storage time, resembling phasic time periods after restorations in simulated natural oral environment; group 1: 24 h, group 2: 3 months, group 3: 6 months.

### 2.2 µTBS test

The exact cross-sectioned areas on surfaces of cement-adhesive-dentin beams were measured by a vernier caliper before micro-tensile bond strength (µTBS) test. Each specimen was then attached to the testing jig of a Motorized Vertical Single-Column test machine (WDD–200–500; WD, China) with instant glue (Aron Alpha Gel–10; Toagosei, Japan), and was subjected to µTBS test at a crosshead speed of 1 mm/min until fracture. Load at failure was recorded and micro-tensile bond strength was calculated. Due to the fan-shaped distribution of the outer dentin tubules at the dentin margin, the portion against the outer side is not recommended even if it does not contain enamel.

$$µ\text{TBS }\left(\text{MPa}\right)=\frac{\text{Maximum}\;\text{load}\;\left(N\right)}{\text{specimens}\;\text{cross}-\text{section}\;\text{areas}\;\left(\text{mm}2\right)}$$

To determine the failure mode, all samples were instantly examined after fracture. Fracture interfaces in group 1 and group 3 were collected and attached to an iron framework. Gold particles had to be coated for 150 s on the surfaces where images to be taken. The de-bonded dentin surfaces were captured by SEM (Quanta 250 FEG; FEI, Czech Republic) at a magnification of ×10000, and were then observed by a microscope (G600; Gaosuo, China). The failure modes were classified by the exact fracture position as (A) complete adhesive failure at the interface between resin cement and dentin; (B) mixed failure (adhesive failure and cohesive failure in the resin cement or in the dentin); (C) cohesive failure in resin cement; (D) cohesive failure in dentin.

### 2.3 Statistical analysis

Comparisons of micro-tensile bonding strength between variables were performed by SPSS program for Windows (Version 19.0). Shapiro-Wilk test was applied to analyze the normality of the data distribution (p > 0.05), while homogeneity amongst variances of the related data were demonstrated by Levene’s test (p > 0.05). The data were subsequently analyzed by three-way ANOVA and Games-Howell test at a 95% level of confidence. The factors were resin cements, curing modes and water storage time.

## 3 Results

### 3.1 µTBS results and statistical analysis

The SPSS analysis results are listed in Table 4. Three-way ANOVA demonstrated that µTBS values were significantly influenced by brand (p < 0.001, F = 59.507), curing mode (p < 0.001, F = 1,237.510), and storage time (p < 0.001, F = 97.888). In addition, a statistical distinction was observed between 2 factors among the 3 variables: brand * curing mode (p < 0.001, F = 56.291), brand * time (p < 0.001, F = 4.931), and curing mode * time (p < 0.001, F = 44.680). The interaction between the above 3 factors was proved to be significant.

**TABLE 4:** Three-way ANOVA analysis result.

Source	df	Mean square	F	P
Correct model	41	797.423	56.133	0.000
Intercept	1	387352.755	27266.961	0.000
brand	6	845.349	59.507	0.000
curing mode	1	17579.990	1237.510	0.000
time	2	1390.586	97.888	0.000
brand * curing mode	6	799.660	56.291	0.000
brand * time	12	70.051	4.931	0.000
curing mode * time	2	634.716	44.680	0.000
brand * curing mode * time	12	29.424	2.071	0.017

The mean and standard deviation values for µTBS were provided in Table 5. Following the light-curing process, the mean TBS values observed in the 24 h groups of all samples were significantly higher than those observed in the aged (3 m and 6 m) groups. No statistically significant differences were observed between the 3 m and 6 m groups, with the exception of RU–S. The 3 m groups exhibited significantly higher µTBS values than the 6 m groups in RU–S. In the self-curing groups, no statistical interaction was evident between different storage times in TC, R2, and N3. A significant difference in µTBS values was noted between the 24 h and 3 m groups in DU, MS, RU, and RU–S. Particularly, the DU group displayed an increasing trend of bonding strength, different from MS, RU, and RU–S groups.

**TABLE 5:** Micro-tensile bond strength (μTBS) values between dual curing resin cement and dentin under different cure modes and after different water storage.

Water storage time	Curing mode	DU	TC	MS	R2	N3	RU	RU–S
24 hours	L	29.94 ± 3.52^A,1^	24.86 ± 7.02^A,2^	31.59 ± 5.35^A,14^	34.35 ± 5.14^A,3^	33.47 ± 6.48^A,34^	34.74 ± 5.32^A,3^	34.94 ± 4.25^A,3^
		*	*	*	*	*	*	*
	D	17.24 ± 3.47^a,12^	17.70 ± 3.22^a,12^	19.44 ± 1.96^a,12^	16.98 ± 1.74^a,2^	26.84 ± 2.21^a,3^	19.62 ± 2.95^a,1^	13.16 ± 3.57^a,4^
3 months	L	22.13 ± 2.95^B,1^	19.65 ± 3.32^B,2^	25.38 ± 2.61^B,3^	29.06 ± 3.51^B,4^	28.64 ± 2.94^B,45^	27.17 ± 4.45^B,34^	26.23 ± 2.69^B,35^
		p > 0.05	p > 0.05	*	*	*	*	*
	D	20.08 ± 3.18^b,1^	17.95 ± 2.94^a,12^	16.20 ± 3.34^b,23^	16.48 ± 4.08^a,23^	25.07 ± 3.01^a,4^	14.96 ± 3.80^b,3^	9.57 ± 5.37^b,5^
6 months	L	22.03 ± 3.40^B,1^	21.72 ± 2.70^B,1^	26.53 ± 2.23^B,23^	27.48 ± 2.99^B,23^	27.69 ± 4.48^B,3^	25.20 ± 3.23^B,24^	23.80 ± 3.67^C,14^
		p > 0.05	*	*	*	p > 0.05	*	*
	D	21.00 ± 3.28^b,1^	19.05 ± 2.67^a,12^	16.57 ± 3.32^b,34^	18.44 ± 4.41^a,13^	25.49 ± 1.96^a,5^	14.70 ± 2.94^b,4^	7.54 ± 4.99^b,6^

Within the same column, the values with different capital superscript among (/L) groups are statistically different (p < 0.05), and different lowercase superscript indicates groups among (/D) with significant difference.

Within the same water storage time and the same column, * between two rows means statistically different values (p < 0.05), and “p > 0.05” between two rows means values are not statistically different (p > 0.05).

Within the same row, the values with different numbers are statistically different (p < 0.05).

As illustrated in Table 6, the mean μTBS values of all resin cements were evaluated according to storage time. A significant difference in μTBS values was evident between 24 h and 3 m, 24 h and 6 m groups among DU, MS, R2, N3, RU, with the former exhibiting notably higher levels of bonding strength. In MS group, the statistical difference was only observed between the 24 h and 3 m groups. Furthermore, a pronounced statistical discrepancy was identified in the 24 h/3 m, 24 h/6m, and 3m/6 m within the RU–S group. When evaluating the bonding strength in relation to prolonged storage, N3 exhibited a markedly elevated bonding strength compared to the remaining groups. Conversely, RU–S demonstrated the worst bonding performance after 3 m and 6 m water storage.

**TABLE 6:** The mean μTBS values of all resin cements in groups of different storage time (mean ± SD).

Water storage time	DU	TC	MS	R2	N3	RU	RU–S
24 h	23.59 ± 7.30^A,1^	21.28 ± 6.49^A,2^	25.51 ± 7.33^A,3^	25.67 ± 9.58^A,3^	30.16 ± 5.83^A,4^	27.17 ± 8.76^A,3^	24.05 ± 11.71^A,13^
3 months	21.11 ± 3.20^B,12^	18.80 ± 3.21^B,3^	20.79 ± 5.51^B,1^	22.77 ± 7.40^B,2^	26.86 ± 3.44^B,4^	21.06 ± 7.41^B,12^	17.90 ± 9.43^B,3^
6 months	21.52 ± 3.34^B,12^	20.38 ± 2.98^AB,1^	21.55 ± 5.77^B,12^	22.96 ± 5.90^B,2^	26.59 ± 3.59^B,3^	19.95 ± 6.13^B,1^	15.67 ± 9.31^C,4^

Within the same column, the values with different capital superscript are statistically different (p < 0.05).

Within the same row, the values with different numbers are statistically different (p < 0.05).

### 3.2 Failure mode

**FIGURE 2:**
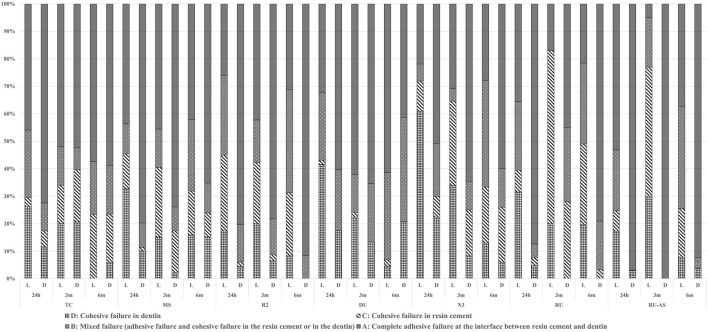
Failure modes of all experimental groups.

Failure modes were depicted in Figure 2. The failure modes revealed a predominant occurrence of elevated A-mode (complete adhesive failure at the interface between resin cement and dentin) across all testing groups. However, notable differences were observed in RU/3 m/L and RU–S/3 m/L, where C-mode (cohesive failure in resin cement) emerged as the primary failure mode. Under dark conditions, A-mode and C-mode exhibited a more pronounced dominance among the failure modes than in light condition. When compared with RU, A-mode presented the predominant failure mode in RU–S, unlike RU groups where the probability of occurrence of each failure mode was more balanced.

### 3.3 SEM

**FIGURE 3:**
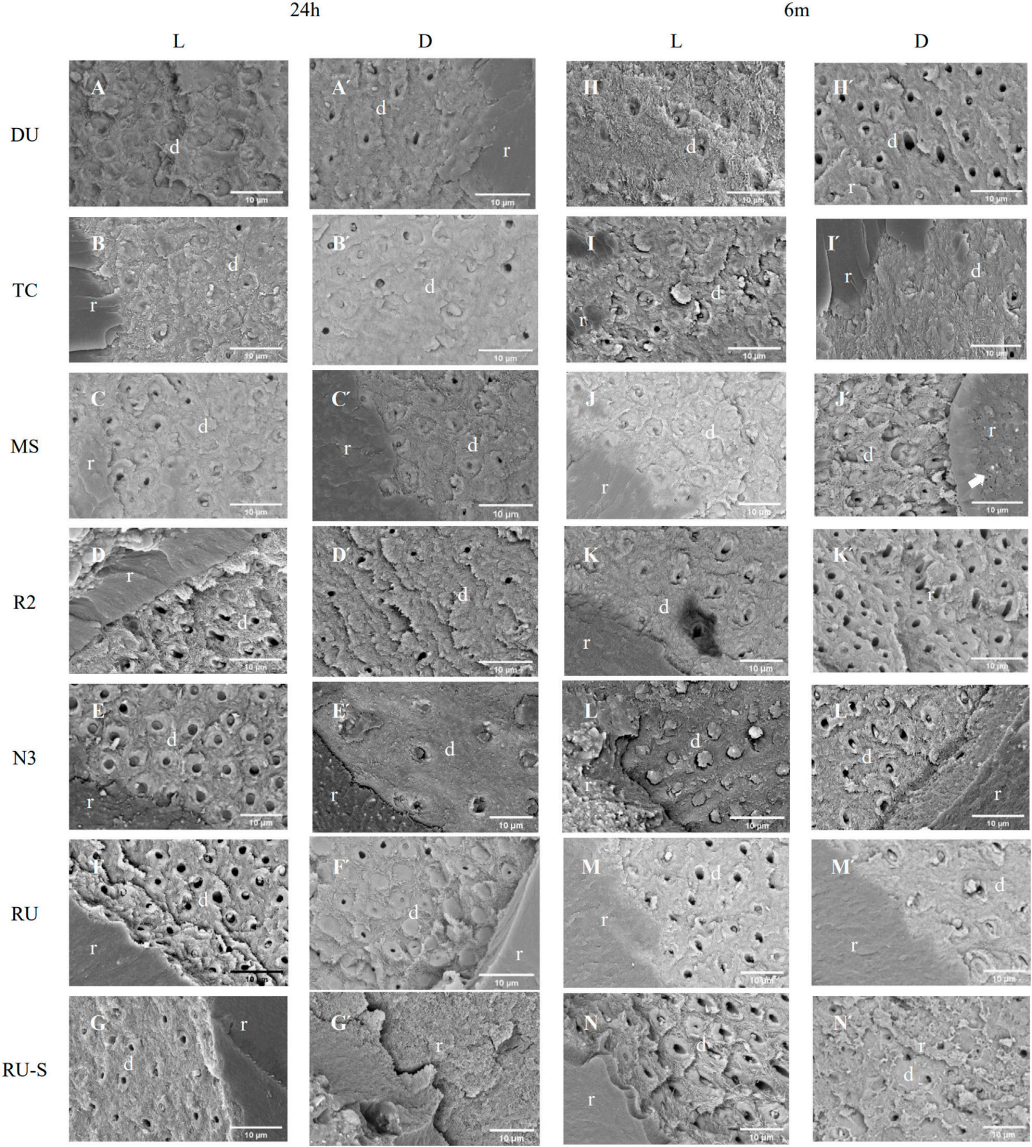
SEM photomicrograph of the fractured surface on the dentin side of samples after 24 hours and 6 months of water storage. Pictures designated by capital letters (A -N and Á -Ń) were at higher magnification (×10000). (A -G) and (Á -Ǵ) correspond to different resin cements with light-curing process after 24 hours and 6 months of water storage, respectively. (H -N) and (H́ -Ń) correspond to different resin cements with self-curing process after 24 hours and 6 months of water storage, respectively. The arrows in both J́ and Ń indicated the presence of voids within resin cement composite or at the surface of resin/dentin. (d = dentin; r = resin cement).

SEM images (magnification ×10,000) of de-bonded cross-sectioned areas of each specimen beam after 24 h and 6 months of water storage were presented in Figure 3. In images A–G (24 h/L), the residual fragments of resin cement were observed, as well as exposure of denuded collagen fibrils on the surface of demineralized dentin following acid-etching treatment. Most dentin tubules were filled with resin cement, indicating a favourable interaction between luting materials and dentin. The fracture interface exhibited either resin cement filling the dentin tubules (E) or traces of cement within them (C, D, F, G). In images Á–Ǵ (24h/D), varying degrees of bonding defects were observed, with open dentinal tubules lacking resin impregnation. A complete cohesive failure within the resin cement was identified in Ǵ (RU–S/24h/D), suggesting incomplete curing. Fewer bonding defects were noted in É (N3/24h/D), where a significant amount of resin cement residue was present within the dentin tubules.

In images H–N (6 m/L), the interface appeared rougher and the remaining resin cement seemed granular, fragmented, and incomplete compared to A–G (24 h/L). In image L (N3/6 m/L), a substantial amount of resin cement covering the dentin tubules with minimal detachment was still evident. In images H́–Ń (6m/D), more empty dentin tubules were observed, indicating incomplete curing or complete debonding, with no signs of resin impregnation. The image reveals uneven surfaces with porous agglomerates (white arrow), suggesting the occurrence of unexpected side reactions that interfered primary polymerization.

## 4 Discussion

The dual curing resin cement has gained considerable acceptance in clinical practice due to its ability to reduce chairside time and achieve the bonding of opaque indirect restorations (Aldhafyan et al., 2021). The ability of self-curing components to achieve the same performance as light-curing and the durability of each resin cement have been the focus of considerable interest (Mazzitelli et al., 2023). Consequently, our experiment was conducted on diverse resin cements, disparate curing modes, and varied water storage times as variables. In order to explain the results, it was necessary to employ the μTBS test, SEM observation, and fracture mode analysis as evidence.

While some resin cements exhibited no statistically significant differences at specific water storage times and curing modes, the overall data illustrated revealed notable intergroup differences. For instance, at the condition of 24 h/L, no significant differences were observed between the R2, N3, RU, and RU-S groups. Nevertheless, a statistical difference was observed in the condition of both 24h/D and 6 m/L among the four groups mentioned above. In conclusion, the experimental results partially rejected hypothesis 1 that each resin cement performs the same bonding performance.

Among all resin cements, N3 exhibited significantly higher bonding strength, both in dark and light conditions and over different storage times, probably owing to its amine-free composition. Tested dual curing resin cements were all combined with specific universal adhesives in our study. Nevertheless, unexpected acid-base interactions arise between the unpolymerized acidic monomers on the surface of the oxygen inhibited layer of a universal adhesive and the tertiary amine component of the resin cement (Tay et al., 2003), thereby interfering with the formation of an interconnected polymer network and the potential achievement of mechanical properties (Dewaele et al., 2009). The absence of amine components in N3 therefore achieved a markedly enhanced bond strength and a notable increase in long-term durability. MS and R2, which represented as both self-adhesive and self-curing resin cement, can be used as luting agents for indirect restorations in the oral cavity (Thammajaruk et al., 2022; Fehrenbach et al., 2021). Previous studies have demonstrated that the bonding strength between the restoration and the treated tooth had been significantly enhanced when universal adhesive was employed at the interface (Santander-Rengifo et al., 2024; Lu et al., 2019). Furthermore, there is no significant statistical difference between TC/D and R2/D after specific water storage time. This is likely due to their ionic composition, including fluoride ions, calcium phosphate and magnesium compounds. The addition of ionic components contributes to a higher bonding strength and enhanced durability (AlSahafi et al., 2022; Liu et al., 2024a). According to statistical analysis of μTBS results, significant interactions were observed except R2/L and N3/L, R2/L and RU/L, RU/L and RU-S/L, RU/D and MS/D.

All specimens except DU/3m, DU/6m, TC/3m, N3/6 m groups pronounced significantly higher µTBS values in dual curing than self-curing experimental groups. Furthermore, the dual curing results in DU/24h, TC/24 h and N3/24 h groups demonstrated a significantly higher µTBS result than corresponding self-curing groups. The μTBS results were in accordance with the failure modes analysis. The percentage of C-mode in self-curing groups was significantly higher than that in dual curing groups, indicating incomplete polymerization of the resin cement, thus resulting in potential appearance of fragile regions. Previous studies have demonstrated that light curing offers significant advantages over self-curing (Vrochari et al., 2009). On the one hand, acidic monomers of self-curing system appear to react with amine initiator components in the dual curing mode, leading to incomplete polymerization (Vrochari et al., 2009). On the other hand, the samples were stored in an oral-simulated moist environment for 24 h after luting. Based on previous studies, self-curing reaction proceeds slowly, and the insufficient polymerized resin cement seemed to be over-sensitive to humidity (Lührs et al., 2014a), resulting in its lower polymerization rate than that of dual curing resin cement (Arrais et al., 2009). The differences in bonding strength between dual curing and self-curing diminished over time in the DU/3m, DU/6m, TC/3m, N3/6 m groups. This result is partially in accordance with previous studies (Carek et al., 2022), which speculated that the potential mechanical properties of the fully cured materials are not accomplished instantly after luting, but develop incrementally, and that the chemical initiator system in dual curing resin cement offers a gradual initiation rate (Arrais et al., 2009; Aguiar et al., 2015). To summarize, our experimental results fail to support hypothesis 2, that the curing modes and storage condition does not affect the bonding strength of different resin cements.

In order to examine the light-curing effect on adhesives, the light curing of the adhesive layer in SBU was omitted in RU-S group. This served as a contrast to RU group, which accomplished the complete operations in accordance with the instructions. According to the instructions provided by the manufacturers, the light-curing steps may be omitted when combining SBU with RU, as the specific components in RU are presumably capable of initiating the self-curing process of SBU. This could result in a reduction in chairside time. The results of μTBS test demonstrated that RU-S/L exhibited significantly higher bonding strength in each water storage time compared with RU-S/D, in conformity with previous studies (Aldhafyan et al., 2022b). Separately applying light curing on the adhesive was supported in early studies, as a forward formation of a relatively stable adhesive layer help sealing dentin surface, thus avoiding water absorption from dentin by micro-leakage in both direct and indirect restorations (Lührs et al., 2014b). The resin cement was placed directly on the adhesives without undergoing light curing. Unexpected reactions occurred between numerous acidic monomers in the adhesives and the tertiary amine in RU, resulting in incomplete chemical polymerization and inadequate intensity (Lu et al., 2019; Liu et al., 2024b). However, no significant difference was observed between RU-S/L and RU/L. In RU-S/L, the application of curing light to the first 2 mm layer of translucent resin cement could result in possible light penetration, initiating the chemical curing process of SBU and achieving adequate bonding strength. In a clinical setting, the use of SBU combined with RU for indirect restoration luting is expected to eliminate the need for independent light-curing steps.

## 5 Conclusion

Within the present study, the following conclusions can be revealed:

1. The bonding strength and durability of dual-cure resin cements were significantly improved by light-curing process.
2. The bonding strength of resin cements with different experimental condition was material depended.
3. Among all tested resin cements, N3 proved to exhibit the most favorable experimental outcomes.

## Data Availability

The original contributions presented in the study are included in the article/supplementary material, further inquiries can be directed to the corresponding author.
